# Association between Oxidative DNA Damage and Risk of Colorectal Cancer: Sensitive Determination of Urinary 8-Hydroxy-2′-deoxyguanosine by UPLC-MS/MS Analysis

**DOI:** 10.1038/srep32581

**Published:** 2016-09-02

**Authors:** Cheng Guo, Xiaofen Li, Rong Wang, Jiekai Yu, Minfeng Ye, Lingna Mao, Suzhan Zhang, Shu Zheng

**Affiliations:** 1 Cancer Institute (Key Laboratory of Cancer Prevention and Intervention, China National Ministry of Education, Key Laboratory of Molecular Biology in Medical Sciences, Zhejiang Province, China), The Second Affiliated Hospital, Zhejiang University School of Medicine, Hangzhou, Zhejiang 310009, China; 2 Department of Gastrointestinal Surgery, Shaoxing People’s Hospital, Shaoxing Hospital of Zhejiang University, Shaoxing, Zhejiang 312000, China; 3 International Health Care Center, The Second Affiliated Hospital, Zhejiang University School of Medicine, Hangzhou, Zhejiang 310009, China

## Abstract

Oxidative DNA damage plays crucial roles in the pathogenesis of numerous diseases including cancer. 8-hydroxy-2′-deoxyguanosine (8-OHdG) is the most representative product of oxidative modifications of DNA, and urinary 8-OHdG is potentially the best non-invasive biomarker of oxidative damage to DNA. Herein, we developed a sensitive, specific and accurate method for quantification of 8-OHdG in human urine. The urine samples were pretreated using off-line solid-phase extraction (SPE), followed by ultrahigh performance liquid chromatography-tandem mass spectrometry (UPLC-MS/MS) analysis. By the use of acetic acid as an additive to the mobile phase, we improved the UPLC-MS/MS detection of 8-OHdG by 2.7−5.3 times. Using the developed strategy, we measured the contents of 8-OHdG in urine samples from 142 healthy volunteers and 84 patients with colorectal cancer (CRC). We observed increased levels of urinary 8-OHdG in patients with CRC and patients with tumor metastasis, compared to healthy controls and patients without tumor metastasis, respectively. Additionally, logistic regression analysis and receiver operator characteristic (ROC) curve analysis were performed. Our findings implicate that oxidative stress plays important roles in the development of CRC and the marked increase of urinary 8-OHdG may serve as a potential liquid biomarker for the risk estimation, early warning and detection of CRC.

Reactive oxygen species (ROS) are generated during normal cellular metabolic processes and after exposure to harmful environmental factors, such as ionizing radiation and chemical carcinogens^1^,^2^,^3^. A large variety of ROS are known in biological systems, the most relevant being hydroxyl radical, hydrogen peroxide, superoxide anion and singlet oxygen. An imbalance between the production and scavenging of ROS, known as oxidative stress, will lead to the damage of cellular proteins, lipids and nucleic acids^4^,^5^. Among these cellular biomolecules that may be attacked and modified by ROS, major efforts have been devoted to DNA^6^,^7^,^8^. It has been recognized that oxidative DNA damage can result in cytotoxic effects and is implicated in the pathogenesis of numerous diseases including neurodegenerative diseases, cardiovascular diseases, chronic inflammatory diseases and cancer^9^,^10^,^11^,^12^.

Although many types of oxidative DNA damage products have been identified^13^, 8-hydroxy-2′-deoxyguanosine (8-OHdG) derived from hydroxyl radical attack of deoxyguanosine residues has been commonly chosen as a biomarker of oxidative damage to DNA. The presence of 8-OHdG residues in DNA results in GC to TA transversion unless repaired prior to DNA replication^14^. Therefore, the presence of 8-OHdG in cells may lead to mutagenesis and thus play a significant role in carcinogenesis and other disease processes^15^. In addition, 8-OHdG may have other detrimental effects on cell function, including reduction of microsatellite stability and acceleration of telomere shortening^16^.

As a matter of fact, persistent oxidative stress exists in cancer^17^. Previous studies have revealed that precancerous and cancerous tissues or cancer cell lines contained 8-OHdG at elevated levels when compared to adjacent normal tissues or to normal cell lines^18^,^19^,^20^,^21^,^22^. For instance, the levels of 8-OHdG in DNA isolated from gastric carcinoma tissue were significantly higher than that from normal gastric tissue, and consistent results were obtained in peripheral blood mononuclear cells of cancer patients compared with healthy controls. Additionally, reduction of urinary 8-OHdG level in cancer patients after surgical operation was also observed^22^. All of these findings prompt us to propose that 8-OHdG is a potential biomarker for cancer risk estimation, early detection, treatment and prognosis.

In the past decades, 8-OHdG has been extensively characterized in cells and tissues in humans and experimental models^18^,^19^,^20^,^21^,^22^,^23^. However, the invasiveness of sample collection and the risk of artifactual oxidation during DNA extraction and subsequent digestion^24^ have limited its application in large-scale human studies. It is generally accepted that the 8-OHdG produced during repairation of oxidatively damaged cellular DNA is excreted into the urine without further metabolism^25^, and the influences of diet and cell turnover on urinary 8-OHdG levels are negligible^26^. These indicate that the level of 8-OHdG in urine could be a good indicator of oxidative damage to DNA in the whole body. Although some urinary chemical constituents are affected by diet, exercise, hormone status and other physiological conditions, and high concentrations of urea and inorganic salts (chloride, sodium, and potassium) in urine may result in ionization source contamination and ion suppression in mass spectrometry detection, compared with other biological matrices such as plasma, serum and saliva, urine has long been considered to be a preferred diagnostic biofluid in clinical practice since it is sterile, easily obtainable in large volumes, and noninvasive to patients^27^,^28^,^29^,^30^,^31^,^32^. Therefore, urinary 8-OHdG analysis should be the first choice for disease risk evaluation, early detection, treatment and prognosis.

A number of analytical techniques for measurement of urinary 8-OHdG have been developed, including enzyme-linked immunosorbent assay (ELISA)^33^,^34^,^35^, capillary electrophoresis with various detectors (e.g., electrochemical or ultraviolet detection, CE-ECD or CE-UV)^36^,^37^,^38^, high-performance liquid chromatography with electrochemical detection (HPLC-ECD)^39^,^40^, gas chromatography-mass spectrometry (GC-MS)^41^,^42^,^43^, and liquid chromatography-tandem mass spectrometry (LC-MS/MS)^33^,^34^,^44^,^45^,^46^,^47^,^48^,^49^,^50^,^51^. ELISA is the most convenient method and has received widespread use, but lack of specificity seems to be the main problem and several reports have demonstrated that ELISA frequently overestimates urinary 8-OHdG levels compared with the concentrations measured by chromatography-based techniques. CE affords high separation efficiency with small sample volumes, but has inherent disadvantages concerning concentration sensitivity and limits of detection. HPLC-ECD requires extensive multi-dimensional chromatography and thorough sample cleanup to reduce the risk of overlapping peaks, although the electrochemical detector is fairly cheap. GC-MS has suffered from a serious artifactual oxidation during chemical derivatization, and thus requires HPLC purification of 8-OHdG before analysis. LC-MS/MS is of great interest due to its advantages in specificity, repeatability, accuracy and structural characterization. Owing to the strong polarity of 8-OHdG, high percentage of aqueous mobile phase is usually used to enhance its retention on a reversed-phase column, leading to poor ionization efficiency in MS along with unsatisfactory sensitivity. However, little attention has been paid to the improvement of the detection sensitivity of 8-OHdG in reversed-phase LC-MS/MS. Although hydrophilic interaction liquid chromatography (HILIC) which uses organic solvents of high concentration is a solution to enhance sensitivity by increasing desolvation and reducing surface tension^52^,^53^, reversed-phase LC-MS/MS methods with improved detection sensitivity for 8-OHdG in human urine is desirable.

Colorectal cancer (CRC) is the third most common cancer in men and the second in women worldwide^54^. Early detection of CRC is critical for reducing its incidence and mortality. To assess the association between oxidative DNA damage and risk of CRC, here we developed an enhanced ionization method for highly sensitive determination of 8-OHdG in human urine by ultrahigh performance liquid chromatography-tandem mass spectrometry (UPLC-MS/MS) analysis combined with a solid-phase extraction (SPE) procedure. By the developed approach, we quantified 8-OHdG in urines from patients with CRC and healthy volunteers. Moreover, logistic regression analysis and receiver operator characteristic (ROC) curve analysis were also performed to evaluate the potential of urinary 8-OHdG as a liquid biomarker for risk estimation, early warning and detection of CRC.

## Materials and Methods

### Chemicals

HPLC grade methanol (MeOH), 8-hydroxy-2′-deoxyguanosine (8-OHdG), 2′-deoxyguanosine (dG), formic acid (HCOOH), acetic acid (CH_3_COOH), ammonium formate (HCOONH_4_) ammonium acetate (CH_3_COONH_4_), ammonium bicarbonate (NH_4_HCO_3_) were purchased from Sigma-Aldrich (St Louis, MO, USA). Water used throughout all experiments was purified using a Milli-Q water purification apparatus (Millipore, Milford, MA, USA). Isotopically labeled internal standard (IS), [^15^N_5_]8-hydroxy-2′-deoxyguanosine ([^15^N_5_]8-OHdG) was purchased from Cambridge Isotope Laboratories Inc. (Andover, MA, USA). 8-OHdG and [^15^N_5_]8-OHdG were reconstituted in water to a stock concentration of 1 mM and 0.1 mM, respectively. Aliquots were stored at −80 °C until required. When required, these were diluted to 1 μM in water.

### Sample collection

This study was approved by the Institutional Review Board of Medical Research, The Second Affiliated Hospital, Zhejiang University School of Medicine (SAHZU) and all experiments were carried out in accordance with the approved guidelines. All of the patients with colorectal cancer (CRC) were pathologic confirmed and had not been treated with surgical operation and chemotherapy. Those individuals were recruited from the Department of Surgical Oncology and healthy volunteers were recruited from International Health Care Center at SAHZU. All subjects gave written informed consent prior to participation.

A total of 84 CRC patients (45 males and 39 females with a mean age of 63.2 ± 12.5 years, range 25–87 years) with TNM (tumor-nodes-metastasis) stage I (n = 14), stage II (n = 26), stage III (n = 34), and stage IV (n = 10) and 142 healthy subjects (74 males and 68 females with a mean age of 46.8 ± 12.1 years, range 21–86 years) were recruited (Table S1). All subjects were asked to provide mid-stream early-morning urine specimens. After urine collection the samples were frozen immediately and stored at −80 °C in the dark until analysis. In order to provide a correction factor for urine concentration, aliquots of urine supernatant were also assayed for creatinine^55^ (Department of Laboratory Medicine, SAHZU).

### UPLC-ESI-MS/MS analysis

The UPLC analysis was performed on an Acquity UPLC system (Waters, Milford, MA, USA), equipped with a binary solvent manager, an autosampler and a column heater. The column was an Acquity UPLC BEH C18 (2.1 mm × 100 mm, 1.7 μm particle size, Waters) maintained at 40 °C. The mobile phase was (A) 0.1% acetic acid, and (B) methanol. An isocratic mode was used to achieve the desired sample separation using 92.5% A and 7.5% B with a flow rate of 0.25 mL/min. Samples were maintained at 4 °C throughout. Each sample was analyzed at least three times with an injection volume of 5 μL. The flow from the column was directed to the mass spectrometer. To optimize the MS detection of 8-OHdG, other four volatile additives to the mobile phase A were tested, including HCOOH, HCOONH_4_, CH_3_COONH_4_, and NH_4_HCO_3_.

The MS detection was performed on a 4000 QTRAP mass spectrometer (AB SCIEX, Foster City, CA, USA) equipped with an ESI ion source (Turbospray) operated in positive ion mode. Instrument control, data acquisition, and processing were performed using the associate Analyst 1.5.2 software. Firstly, collision-induced dissociation (CID) experiment^56^ of 8-OHdG standard was performed in product ion scan mode and the spectrum was illustrated in Figure S1. Then MS/MS data were acquired in multiple reaction monitoring (MRM) mode. Two transitions between precursor ion and the two most abundant product ions were monitored: the transition *m/z* 284.1 > 168.0 for quantitative determination and the transition *m/z* 284.1 > 117.0 for qualitative analysis. And for [^15^N_5_]8-OHdG the transition *m/z* 289.1 > 173.0 was monitored for quantitative determination. To increase sensitivity, the ion source temperature (TEM) was set at 550 °C, and the ion spray voltage (IS) was set at 5.5 kV. Ion source gas 1 (GS1) and ion source gas 2 (GS2) used as the nebulizing and drying gases were set at 60 and 40 psi, respectively. Curtain gas (CUR) was set at 35 psi. The optimized MS conditions used for the analysis of the target analyte were shown in Table S2.

### Solid-phase extraction

The urine samples were fully thawed on the day of extraction at room temperature and centrifuged at 13000 rpm for 15 min at 4 °C. 500 μL of supernatant was diluted 1:1 with water, and then spiked with 10 pmol of [^15^N_5_]8-OHdG internal standard. The samples were pretreated using Oasis HLB (3.0 mL, 60 mg) cartridges (Waters, Milford, MA, USA). Each cartridge was preconditioned with 1.0 mL of methanol followed by 1.0 mL of water, and then urine samples were loaded. The cartridges were washed with 1.0 mL of methanol/water of 5:95 (v/v), followed by the final elution using 1.0 mL of methanol/water of 1:1 (v/v). The eluted fractions were evaporated under vacuum. The dried urine samples were reconstituted in 200 μL of water for UPLC-MS/MS analysis.

### Validation of analytical method

Method performance was investigated with respect to various parameters such as selectivity, linearity, limit of detection (LOD), limit of quantification (LOQ), precision, accuracy, recovery, and matrix effect.

The selectivity of the method was conducted in the MRM mode. For the analyte, two transitions were monitored. The more abundant one was used for quantification and the other one for confirmation. Besides, the selectivity was also tested by visual inspection of chromatograms of extracted urine samples for the presence of endogenous interfering peaks.

The working solutions of 8-OHdG standard with increasing concentrations (1.0, 2.5, 5.0, 10.0, 20.0, 50.0, 100.0, 200.0, 300.0 nM) mixed with 10 pmol of IS solution (final concentration, 50 nM) were prepared and then analyzed using the UPLC-MS/MS method described above. Calibration curve was constructed and expressed as equation *y* = a*x* + b, where *y* stands for the peak area ratio of 8-OHdG to the corresponding IS and *x* standard for the concentration of 8-OHdG. The LOD was determined by analyzing standard solution at levels that provided signals at three times above the background noises. In a similar way, the LOQ was identified at signal to noise ratios equal to ten.

In assessment of the intra- and inter-day precision, quality control samples at three different concentration levels were used, namely 10 nM (low quality control, LQC), 50 nM (medium quality control, MQC) and 200 nM (high quality control, HQC). For intraday precision, three concentration levels were prepared three times simutaneously and each sample was analyzed in three replicates. The total number of measurements for one concentration level was nine. For interday precision, the same procedure was performed in three consecutive days. The accuracy of the method was measured by determining the mean concentration of QC samples and was expressed as a percentage of the theoretical concentration (accuracy = mean observed concentration/theoretical concentration × 100%).

For evaluation of extraction recovery, known amounts of 8-OHdG (5, 20 and 60 nM) were added to the urine samples, respectively. After addition of 10 pmol of IS solution, each sample was passed through a HLB cartridge and analyzed by UPLC-MS/MS as described above. The recovery (R) was determined according to the formula, R = (concentration in spiked sample − concentration in original sample)/spiked concentration × 100%.

Since it is difficult to find blank urine samples without 8-OHdG, the slope comparison method was used to evaluate the matrix effect^57^. Ten aliquots of urine extracts were spiked with standard solutions of increasing concentrations (0.0, 1.0, 2.5, 5.0, 10.0, 20.0, 50.0, 100.0, 200.0, 300.0 nM) and 10 pmol of IS solution. The first aliquot was only IS solution spiked. Then the standard addition calibration curve was constructed and the slope was compared with that obtained from the standard solutions. Matrix effect was calculated as the slope ratio of the standard addition calibration curve to the standard solutions calibration curve.

### Statistical analysis

All the statistical analyses were performed using SPSS statistics 20.0 software (IBM, Armonk, NY, USA). T-test was applied to evaluate the differences of concentration levels of urinary 8-OHdG between normal volunteers and patients with CRC. Concentrations of urinary 8-OHdG in different cancer stages were compared with Oneway ANOVA. Logistic regression model was established to determine the relationship between CRC detection and other factors, such as age, gender and concentration of urinary 8-OHdG. Receiver operator characteristic (ROC) curve analysis was used to evaluate the fitting effect of logistic regression model. Statistical tests were two sided and *p* < 0.05 was considered statistically significant.

## Results and Discussion

### Selection of mobile phase additive to enhance the MS detection of 8-OHdG

**Figure 1:**
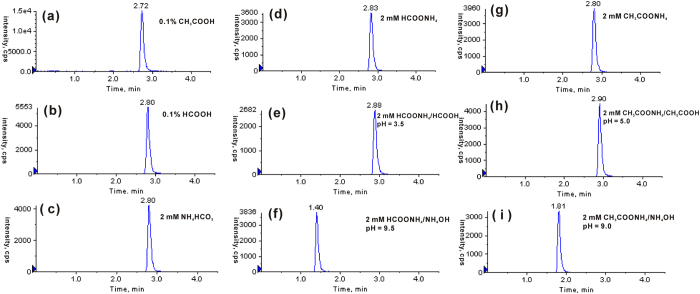
Effect of mobile-phase additives on the MS detection sensitivity of 8-OHdG. The mobile phase consisted of solvents A and B (pure methanol). An isocratic elution of 92.5% A and 7.5% B was used, and the flow was set at 0.25 mL/min. The concentration of 8-OHdG standard was 20 nM, and the injection volume was 5.0 μL.

Mobile phase additive plays important roles not only in chromatographic separation but also in MS detection^57^,^58^. In this work, five volatile additives, including CH_3_COOH, CH_3_COONH_4_, HCOOH, HCOONH_4_ and NH_4_HCO_3_ were chosen and tested as the potential mobile phase additives for LC-ESI-MS/MS analysis of 8-OHdG. As shown in Fig. 1, nine types of mobile phase A were prepared and examined, and the peak area ratios for 8-OHdG obtained by other eight types of mobile phase versus mobile phase with NH_4_HCO_3_ as an additive were summarized in Table 1. Compared with other additives, CH_3_COOH increased the signal response of 8-OHdG by 2.7−5.3 times. Besides, signal-to-noise of the analyte was also elevated with CH_3_COOH as a mobile phase additive.

Moreover, we compared the ionization complex distribution of 8-OHdG using these additives to the mobile phase. A 20 μM 8-OHdG standard solution was used and the UPLC fraction was scanned under the positive ionization mode by ESI-MS. HCOOH, HCOONH_4_, CH_3_COONH_4_ or NH_4_HCO_3_ as the mobile-phase additive promotes 8-OHdG to form abundant [8-OHdG + Na]^+^ and [8-OHdG + K]^+^ ions, while CH_3_COOH as the mobile-phase additive favors the formation of [8-OHdG + H]^+^ ion. The peak area ratios ([M + H]^+^/([M + Na]^+^ + [M + K]^+^)) of 8-OHdG were measured under these different mobile phase conditions. As shown in Table 1, all of the ratios tend to decrease in the presence of other additives compared to CH_3_COOH in the eluent. For instance, the full scan mass spectra of 8-OHdG using CH_3_COOH or CH_3_COONH_4_ as additives were illustrated in Figure S2. When using 0.1% CH_3_COOH as mobile phase A (Figure S2(a)), the signal intensity of [M + H]^+^ ion was higher than that of [M + Na]^+^ ion, and the signal intensity of [M + K]^+^ ion was very low. However, the formation of [M + H]^+^ ion was inhibited and the signal intensity is lower than that of [M + Na]^+^ ion when 2 mM CH_3_COONH_4_ was used (Figure S2(b)). In addition, CH_3_COONH_4_/CH_3_COOH and CH_3_COONH_4_/NH_4_OH buffer systems at pH 5.0 or 9.0 promoted the formation of [M + K]^+^ ion (Figure S2(c,d)). These data suggest that CH_3_COOH improves the ionization efficiency of 8-OHdG by suppressing the formation of 8-OHdG−metal adducts during the ESI process.

Since 2′-deoxyguanosine (dG) might be oxidized to 8-OHdG during ESI process due to the existence of high voltage, chromatographic separation of dG and 8-OHdG was essential to avoid the interference from dG. Fortunately, excellent separation was achieved when using CH_3_COOH as the mobile-phase additive (Figure S3).

### Validation of analytical method

The qualitative ion transition (*m/z* 284.1 > 117.0) was used in the analysis of 8-OHdG to confirm the identity of 8-OHdG peaks especially when concentrations were low. In addition, by comparing the retention time and the MS/MS signals of 8-OHdG in urine samples with those of standard solutions, the target analyte could be easily distinguished and no interference peaks were observed, which indicated a good selectivity of the analytical method.

Calibration curve was constructed by plotting the peak area ratio (*y*) of 8-OHdG to [^15^N_5_]8-OHdG versus the concentration of 8-OHdG (*x*, nM). Excellent linearity was achieved (*y* = 0.0278*x* + 0.0515) in the concentration range from 1.0 to 300.0 nM with the correlation coefficient of R^2^ = 0.9993.

The LOD and LOQ were considered as the analyte minimum concentrations that can be confidently identified and quantified by the method, respectively. The LOD was 1 fmol and the LOQ was 3 fmol, which is consistent with the result obtained by HILIC method^53^. This indicated that the sensitivity of the analytical method developed was excellent.

Precision of the method was evaluated by measuring intra- and inter-day relative standard deviations (RSD). The intraday precision values varied from 1.0% to 1.9%, and the interday precision values varied from 0.9% to 1.4%. The accuracy values of the intraday study and the interday assay were in the range of 98.0–101.7%, which were in an acceptable range ±15% of the theoretical concentration. The results obtained are shown in Table 2, and all these data revealed that the precision and accuracy of the described method were satisfactory.

The recovery was measured by spiking urine samples with known amounts of 8-OHdG (5, 20 and 60 nM, final concentration). At all fortification levels, recoveries were in the range of 101.9–118.1%, with RSD values less than 4.0% (Table 3). The slope ratio value was 91.5% (Figure S4), indicating that the effect of matrix on the ionization efficiency of 8-OHdG was negligible in this study.

In addition, a QC sample was measured every fifteen urine samples to monitor whether the system was still stable after hundreds of injections. The parameters such as retention time, peak symmetry and also accuracy were checked and the results showed good system stability. All these results proved that the developed SPE combined with UPLC-MS/MS method using CH_3_COOH as mobile phase additive was sensitive, accurate and reproducible to analyze 8-OHdG in urine samples.

### Identification and quantification of 8-OHdG in human urine

**Figure 2:**
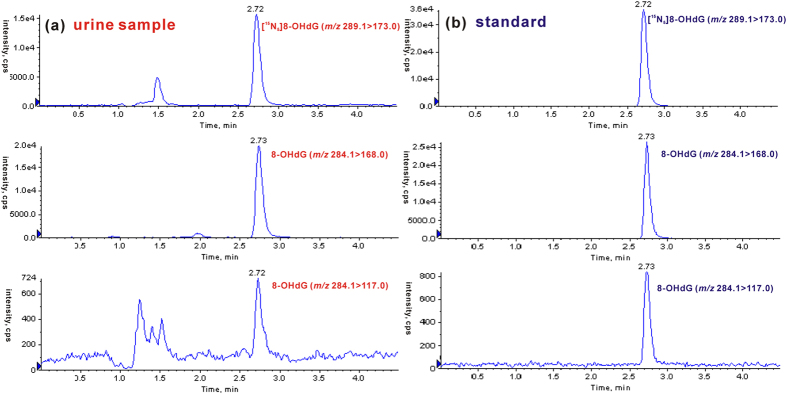
Identification of 8-OHdG in urine sample by UPLC-MS/MS. (**a**) Representative chromatograms from human urine sample displaying internal standard [^15^N_5_]8-OHdG (*m/z* 289.1 > 173.0), 8-OHdG (*m/z* 284.1 > 168.0) and corresponding qualifier ion (*m/z* 284.1 > 117.0). (**b**) Representative chromatograms of internal standard and 8-OHdG standard.

By the developed off-line SPE-coupled UPLC-MS/MS strategy, we further examined 8-OHdG in urine samples from 142 healthy volunteers and 84 patients with CRC. As shown in Fig. 2(a), the retention time of 8-OHdG from human urine is identical to that of the added internal standard ([^15^N_5_]8-OHdG). Moreover, urinary 8-OHdG produces a transition with the second highest abundance (*m/z* 284.1 > 117.0), which is consistent with the nonisotope 8-OHdG standard (Fig. 2(b)). The signal ratio of the quantitative ion transition (*m/z* 284.1 > 168.0) to the qualitative ion transition (*m/z* 284.1 > 117.0) was also evaluated, and the ratio for urinary 8-OHdG is almost the same as that of the nonisotope 8-OHdG standard (~31, evaluated by peak height). These results indicate that urinary 8-OHdG has the same chromatographic retention and the same dissociation pathway upon collisional activation as the 8-OHdG standard, consistently confirming the presence of 8-OHdG in human urine.

**Figure 3:**
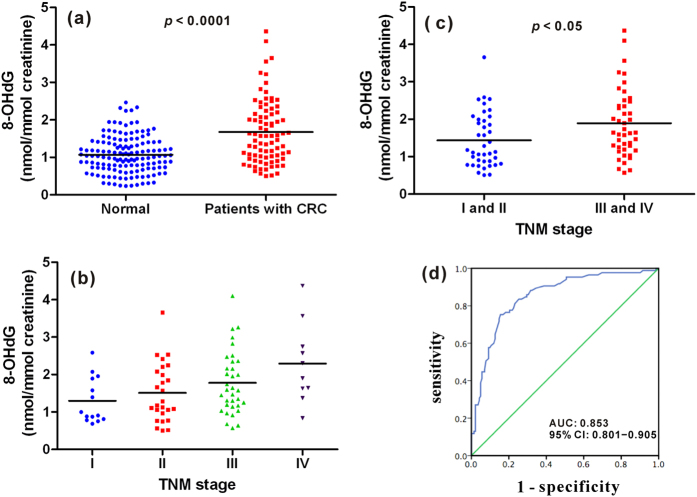
Quantification and statistical analysis of 8-OHdG in human urine samples. (**a**) 8-OHdG content in healthy volunteers and patients with CRC. (**b**) 8-OHdG content in patients from stage I to IV. (**c**) 8-OHdG content in patients without (stage I and II) and with (stage III and IV) tumor metastasis. (**d**) ROC curve for urinary 8-OHdG score.

The urinary 8-OHdG level was normalized with creatinine concentration and is presented as nmol of 8-OHdG/mmol of creatinine. The concentration of all the collected urine samples was illustrated in Fig. 3(a) and the detailed data were listed in Table S1. The measured concentration of 8-OHdG in urine samples from healthy controls is about 0.24−2.47 nmol/mmol creatinine, and the average concentration is about 1.07 ± 0.49 nmol/mmol creatinine (n = 142). The concentration of 8-OHdG in urine from patients with CRC is in the range of 0.51−4.37 nmol/mmol creatinine, and the average concentration is about 1.68 ± 0.85 nmol/mmol creatinine (n = 84). It is evident that the level of urinary 8-OHdG was markedly increased in patients with CRC relative to the healthy controls and there is significant difference (*p* < 0.0001). This indicates that the content of 8-OHdG in human urine may serve as an efficient indicator of CRC.

Additionally, the concentration of urinary 8-OHdG of patients with CRC in different tumor stages was also evaluated to probe the correlation between oxidative DNA damage and the progression of CRC. The stage I and stage II are the tumor limited in the bowel wall, and the stage III is the tumor with localized lymph node metastasis and the stage IV is the tumor with distant metastasis to liver and lung. As shown in Fig. 3(b), the concentration of urinary 8-OHdG elevated gradually from stage I to IV. The average concentration of urinary 8-OHdG of patients in stage I, II, III and IV is 1.30 ± 0.61, 1.51 ± 0.78, 1.78 ± 0.84 and 2.29 ± 1.07 nmol/mmol creatinine, respectively. This indicates that the oxidative damage becomes more serious accompanied with the progression of CRC. Moreover, for patients in stage III and IV with tumor regional and distant metastasis, the concentration of urinary 8-OHdG is significantly higher than that of patients in stage I and II without tumor metastasis (*p* < 0.05, Fig. 3(c)). As a matter of fact, increased oxidative stress during CRC progression from operable CRC to non-operable liver metastasis, as observed by depletion of antioxidant vitamins and enhancement of lipid peroxidation has been demonstrated^59^. These results imply that the urinary 8-OHdG level not only could be used to indicate the presence of CRC, but also assess whether the tumor have metastasized to lymph nodes or distant organs such as liver and lung.

In order to further evaluate the correlation of the development of CRC with respect to urinary 8-OHdG concentration, age and gender, logistic regression analysis was performed. The results demonstrated that individuals with higher concentration of urinary 8-OHdG and older age were more likely to develop CRC, whereas gender was not involved with the development of CRC (Table 4). Compared with individuals whose urinary 8-OHdG concentrations were lower than 1.5 nmol/mmol creatinine, individuals with concentrations higher than 1.5 nmol/mmol creatinine had a 3.68-fold increased risk of developing CRC (odds ratio = 3.68, 95% confidence interval (CI) 1.82−7.45, *p* < 0.0001).

Besides, a logistic regression model consisted of urinary 8-OHdG concentration and age was established to predict CRC risk, and the probability was determined according to the formula shown below.


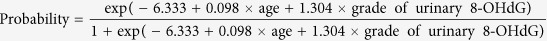


The value of grade of urinary 8-OHdG is set at 1 or 0 when the urinary 8-OHdG concentration is higher or lower than 1.5 nmol/mmol creatinine, respectively. The cutoff value of this model was 0.5, which meant that individuals with a probability greater than 0.5 had significantly higher risk of developing CRC than those with a probability less than 0.5. On the other hand, a logistic regression model only consisted of urinary 8-OHdG concentration was established and ROC analysis was performed. As illustrated in Figure S5, the area under the curve (AUC) is 0.722, 95% CI 0.652−0.791, *p* < 0.0001. And for the logistic regression model consisted of urinary 8-OHdG concentration and age, the ROC curve indicated good fitting effect of this logistic regression model and a bigger AUC value which implies more effective detection was obtained. As shown in Fig. 3(d), 8-OHdG was highly effective in the detection of CRC with AUC being 0.853, 95% CI 0.801−0.905, *p* < 0.0001.

So far, biomarkers including carcino-embryonic antigen (CEA) routinely used for clinical detection of CRC are measured in serum, all invasive to human beings. It is noteworthy that our findings indicate 8-OHdG in urine can be a potential non-invasive liquid biomarker for risk estimation, early warning and detection of human CRC. Moreover, it can also be used to monitor the progressing of CRC.

## Conclusions

In the present study, we developed an off-line SPE-coupled UPLC-MS/MS strategy for accurate quantification of 8-OHdG in urine samples from 142 healthy volunteers and 84 patients with CRC. The use of acetic acid significantly enhanced the UPLC-MS/MS detection of 8-OHdG by 2.7–5.3 times. We demonstrated that the levels of urinary 8-OHdG were markedly increased in patients with CRC relative to the healthy controls. Moreover, for patients with CRC, the content of urinary 8-OHdG concentration elevated gradually from stage I to IV, and for patients with tumor metastasis, the content of urinary 8-OHdG was significantly higher than that in patients without tumor metastasis. Additionally, logistic regression analysis and receiver operator characteristic (ROC) curve analysis were also performed, and the results revealed that urinary 8-OHdG could be utilized in prediction of CRC risk and highly effective detection of CRC. All of these findings suggest that oxidative stress plays important roles in the development of CRC and the marked increase of urinary 8-OHdG may serve as a potential non-invasive liquid biomarker for the risk estimation, early warning and detection of CRC.

## Additional Information

**How to cite this article**: Guo, C. *et al*. Association between Oxidative DNA Damage and Risk of Colorectal Cancer: Sensitive Determination of Urinary 8-Hydroxy-2′-deoxyguanosine by UPLC-MS/MS Analysis. *Sci. Rep.* **6**, 32581; doi: 10.1038/srep32581 (2016).

## Supplementary Material

Supplementary Information

## Figures and Tables

**Table 1:** The peak area ratios of 8-OHdG obtained in different mobile phase A additives conditions.

Mobile phase A additives	Peak area ratios	
	MRM mode^a^	Full scan mode^b^
0.1% CH_3_COOH	3.2	1.0
0.1% HCOOH	1.2	0.7
2 mM NH_4_HCO_3_	1.0	0.4
2 mM HCOONH_4_	0.8	0.4
2 mM HCOONH_4_/HCOOH, pH = 3.5	0.6	0.5
2 mM HCOONH_4_/NH_4_OH, pH = 9.5	0.7	0.5
2 mM CH_3_COONH_4_	0.9	0.5
2 mM CH_3_COONH_4_/CH_3_COOH, pH = 5.0	0.9	0.8
2 mM CH_3_COONH_4_/NH_4_OH, pH = 9.0	0.7	0.6

^a^The peak area ratios of 8-OHdG were obtained by other eight types of mobile phase *vs* mobile phase with NH_4_HCO_3_ as an additive.

^b^The peak area ratios were calculated as peak area of [8-OHdG + H]^+^/(peak area of [8-OHdG + Na]^+^ + peak area of [8-OHdG + K]^+^).

**Table 2:** Precision and accuracy for 8-OHdG QC samples at three different concentrations.

	Levels of 8-OHdG		
	LQC (10 nM)	MQC (50 nM)	HQC (200 nM)
Intraday (n = 9)			
Mean ± SD (nM)	9.80 ± 0.18	50.55 ± 0.59	198.97 ± 2.06
RSD (%)	1.9	1.2	1.0
Accuracy (%)	98.0	101.1	99.5
Interday (n = 3)			
Mean ± SD (nM)	9.85 ± 0.14	50.84 ± 0.73	199.52 ± 1.84
RSD (%)	1.4	1.4	0.9
Accuracy (%)	98.5	101.7	99.8

**Table 3:** Recoveries of the off-line SPE-coupled UPLC-MS/MS obtained at three different spiking levels.

	Added amount of 8-OHdG (nM)			
	0	5 (low)	20 (medium)	60 (high)
Mean ± SD (nM)	18.70 ± 0.39	24.60 ± 0.53	39.08 ± 0.68	82.30 ± 1.04
Average recovery (%)	—	118.1	101.9	106.0
RSD (%)	—	3.3	2.4	1.9

**Table 4:** Logistic regression analysis of factors associated with CRC.

Variable		Odds ratio	95% CI	*p*
Urinary 8-OHdG concentration(nmol/mmol creatinine)	>1.5	3.68	1.82–7.45	<0.0001
	≤1.5	1	—	—
Age		1.10	1.07–1.14	<0.0001
Gender	Male	0.58	0.28–1.18	0.111
	Female	1	—	—
