# Supplementary material for: Association between Oxidative DNA Damage and Risk of Colorectal Cancer: Sensitive Determination of Urinary 8-Hydroxy-2′-deoxyguanosine by UPLC-MS/MS Analysis
Source: Sci Rep. 2016 Sep 2;6:32581. doi: 10.1038/srep32581 (PMC5009303; doi:10.1038/srep32581)
Supplement: Supplementary Information [file srep32581-s1.pdf]

Association between Oxidative DNA Damage and Risk of Colorectal Cancer: Sensitive Determination of Urinary 8-Hydroxy-2'-deoxyguanosine by UPLC-MS/MS Analysis

Cheng Guo[1], Xiaofen Li[1], Rong Wang[1], Jiekai Yu[1], Minfeng Ye[1,2], Lingna Mao[1,3], Suzhan Zhang[1] and Shu Zheng[1*]

[1]Cancer Institute (Key Laboratory of Cancer Prevention and Intervention, China National Ministry of Education, Key Laboratory of Molecular Biology in Medical Sciences, Zhejiang Province, China), The Second Affiliated Hospital, Zhejiang University School of Medicine, Hangzhou, Zhejiang 310009, China

[2]Department of Gastrointestinal Surgery, Shaoxing People's Hospital, Shaoxing Hospital of Zhejiang University, Shaoxing, Zhejiang 312000, China

[3]International Health Care Center, The Second Affiliated Hospital, Zhejiang University School of Medicine, Hangzhou, Zhejiang 310009, China

*Corresponding author: Shu Zheng

Cancer Institute, The Second Affiliated Hospital, Zhejiang University School of Medicine, Jiefang Road 88#, Hangzhou, Zhejiang 310009, China.

Tel: +86-571-87784501; Fax: +86-571-87214404.

E-mail address: zhengshu@zju.edu.cn.

Supplementary information

Content

- **Figure S1. The collision-induced dissociation (CID) mass spectrum of 8-OHdG.**
- **Figure S2. Typical ESI-MS spectra of 8-OHdG when different additives were used.** Additives to mobile phase A: (a) 0.1% CH_3COOH, (b) 2 mM CH_3COONH_4, (c) 2 mM CH_3COONH_4/CH_3COOH, pH = 5.0, and (d) 2 mM CH_3COONH_4/NH_4OH, pH = 9.0 were selected as examples for clarity. An isocratic elution of 92.5% A and 7.5% B was used, and the flow was set at 0.25 mL/min. The concentration of 8-OHdG standard was 20 μM, and the injection volume was 5.0 μL.
- **Figure S3. The chromatographic separation of 8-OHdG and dG.** The mobile phase consisted of solvents A (0.1% acetic acid) and B (pure methanol). An isocratic elution of 92.5% A and 7.5% B was used, and the flow was set at 0.25 mL/min. The concentration of 8-OHdG standard was 20 nM, and the injection volume was 5.0 μL.
- **Figure S4. Calibration curve of 8-OHdG standard obtained in water and human urine.**
- **Figure S5. ROC curve only using urinary 8-OHdG concentration.**
- **Table S1. Clinic information and urinary 8-OHdG concentration of healthy volunteers and patients with colorectal cancer.**
- **Table S2. The optimized MS conditions used for the analysis of 8-OHdG.**

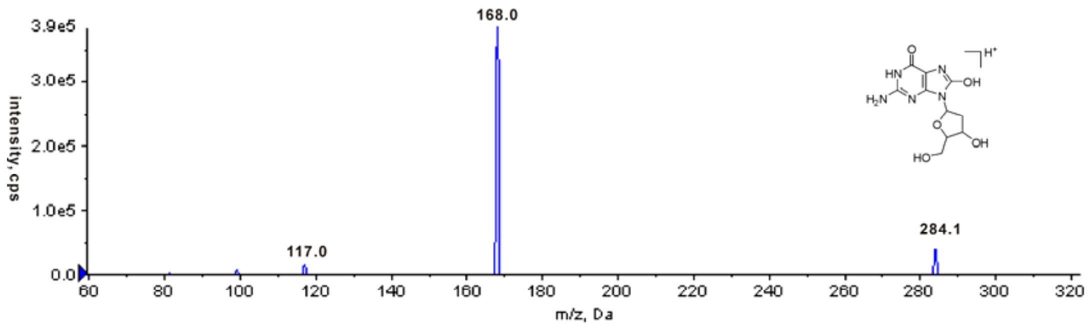

Figure S1. The collision-induced dissociation (CID) mass spectrum of 8-OHdG.

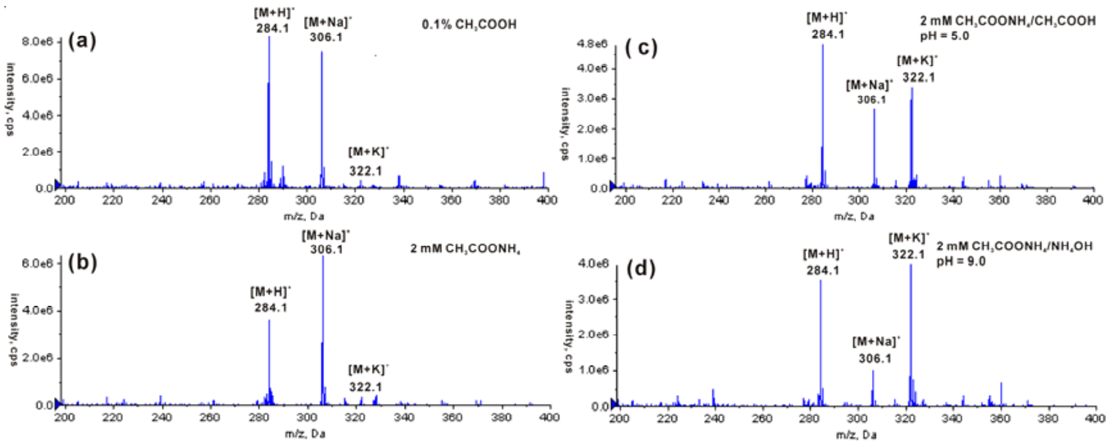

Figure S2. Typical ESI-MS spectra of 8-OHdG when different additives were used. Additives to mobile phase A: (a) 0.1% CH_3COOH, (b) 2 mM CH_3COONH_4, (c) 2 mM CH_3COONH_4/CH_3COOH, pH = 5.0, and (d) 2 mM CH_3COONH_4/NH_4OH, pH = 9.0 were selected as examples for clarity. An isocratic elution of 92.5% A and 7.5% B was used, and the flow was set at 0.25 mL/min. The concentration of 8-OHdG standard was 20 µM, and the injection volume was 5.0 µL.

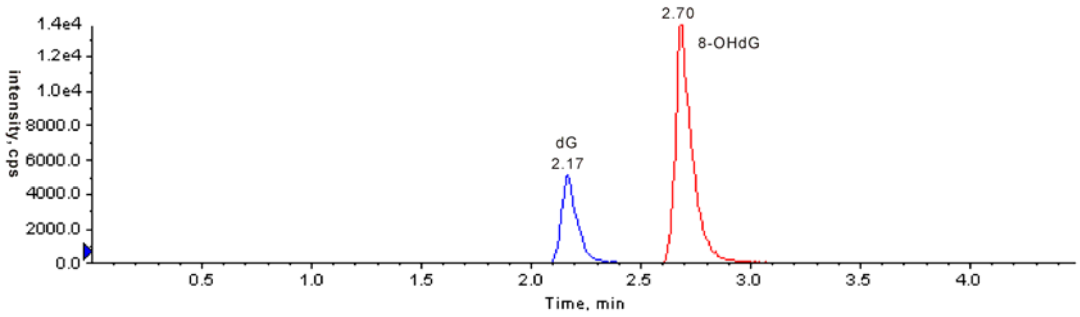

Figure S3. The chromatographic separation of 8-OHdG and dG. The mobile phase consisted of solvents A (0.1% acetic acid) and B (pure methanol). An isocratic elution of 92.5% A and 7.5% B was used, and the flow was set at 0.25 mL/min. The concentration of 8-OHdG standard was 20 nM, and the injection volume was 5.0 μL.

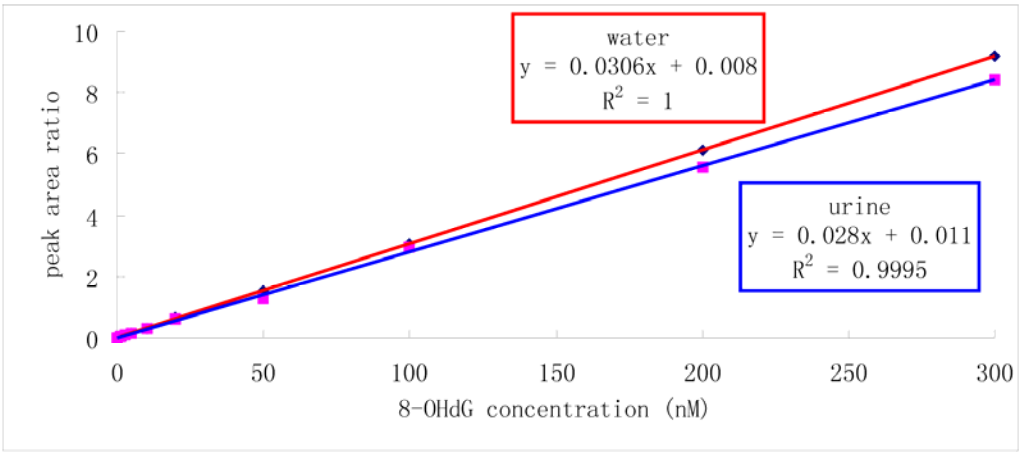

Figure S4. Calibration curve of 8-OHdG standard obtained in water and human urine.

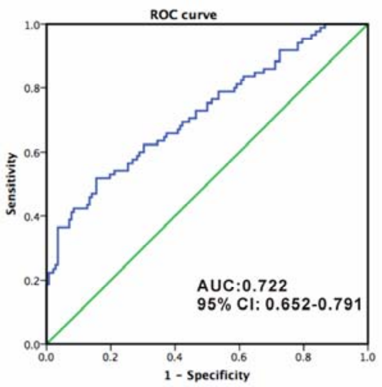

Figure S5. ROC curve only using urinary 8-OHdG concentration.

Table S1. General information and urinary 8-OHdG concentration of healthy volunteers and patients with colorectal cancer.

No.	Age (years)	Sex	Urinary 8-OHdG concentration (nmol/mmol creatinine)
Normal-1	29	M	0.942±0.043
Normal-2	27	F	0.798±0.025
Normal-3	27	F	1.077±0.025
Normal-4	51	F	1.372±0.015
Normal-5	55	F	1.097±0.032
Normal-6	39	M	0.518±0.027
Normal-7	29	M	1.461±0.053
Normal-8	30	M	1.852±0.036
Normal-9	39	M	0.791±0.014
Normal-10	42	M	1.918±0.059
Normal-11	36	F	1.552±0.026
Normal-12	36	M	0.884±0.029
Normal-13	37	M	0.882±0.023
Normal-14	29	M	0.690±0.019
Normal-15	37	M	0.472±0.019
Normal-16	31	M	1.426±0.086
Normal-17	37	M	0.306±0.008
Normal-18	51	F	0.625±0.017
Normal-19	30	F	0.730±0.017
Normal-20	26	M	1.288±0.097
Normal-21	57	M	1.025±0.026
Normal-22	56	M	1.035±0.005
Normal-23	29	F	0.467±0.005
Normal-24	35	M	0.514±0.025
Normal-25	73	M	1.166±0.018
Normal-26	83	M	0.447±0.011
Normal-27	63	M	0.964±0.019
Normal-28	71	M	0.296±0.006
Normal-29	46	F	1.364±0.015
Normal-30	86	M	0.769±0.011
Normal-31	67	M	0.935±0.071
Normal-32	65	M	1.242±0.073
Normal-33	67	M	0.294±0.013
Normal-34	58	M	1.172±0.045
Normal-35	71	M	0.263±0.014
Normal-36	59	M	0.788±0.022
Normal-37	65	M	2.312±0.100
Normal-38	47	M	0.785±0.023

Normal-39	50	F	0.938±0.053
Normal-40	80	M	0.821±0.008
Normal-41	57	M	1.298±0.028
Normal-42	72	M	1.937±0.215
Normal-43	50	F	0.234±0.007
Normal-44	53	M	0.721±0.036
Normal-45	47	F	1.001±0.020
Normal-46	37	M	1.152±0.007
Normal-47	49	M	0.771±0.001
Normal-48	40	M	0.664±0.014
Normal-49	52	F	1.130±0.064
Normal-50	48	M	0.541±0.023
Normal-51	48	M	0.728±0.011
Normal-52	52	M	0.814±0.034
Normal-53	47	M	0.564±0.028
Normal-54	42	M	0.910±0.027
Normal-55	62	F	0.870±0.010
Normal-56	53	M	1.491±0.036
Normal-57	41	F	0.702±0.070
Normal-58	62	M	0.667±0.024
Normal-59	52	M	1.627±0.050
Normal-60	44	F	0.901±0.022
Normal-61	51	F	0.488±0.023
Normal-62	35	F	0.827±0.015
Normal-63	39	F	0.835±0.023
Normal-64	47	F	0.464±0.003
Normal-65	41	F	1.396±0.012
Normal-66	50	F	0.869±0.030
Normal-67	41	F	1.072±0.033
Normal-68	55	F	1.105±0.045
Normal-69	43	F	1.107±0.021
Normal-70	48	F	0.588±0.063
Normal-71	35	F	1.722±0.070
Normal-72	52	F	1.049±0.055
Normal-73	55	F	1.654±0.084
Normal-74	46	F	0.441±0.017
Normal-75	42	F	0.481±0.026
Normal-76	50	F	1.181±0.028
Normal-77	45	F	1.758±0.010
Normal-78	64	F	1.696±0.049
Normal-79	41	F	1.288±0.047
Normal-80	60	F	1.932±0.053
Normal-81	38	F	0.981±0.097
Normal-82	42	F	1.415±0.059

Normal-83	57	F	0.699±0.013
Normal-84	47	F	1.908±0.037
Normal-85	36	F	0.918±0.026
Normal-86	41	F	0.326±0.019
Normal-87	51	F	1.233±0.036
Normal-88	44	F	0.425±0.035
Normal-89	47	M	1.218±0.020
Normal-90	52	M	1.568±0.018
Normal-91	39	F	0.241±0.013
Normal-92	45	F	0.474±0.022
Normal-93	48	F	1.399±0.061
Normal-94	50	M	1.073±0.113
Normal-95	50	F	1.644±0.058
Normal-96	26	F	0.964±0.008
Normal-97	47	F	0.573±0.046
Normal-98	26	F	0.704±0.020
Normal-99	32	F	1.611±0.032
Normal-100	23	F	1.037±0.028
Normal-101	49	F	1.341±0.070
Normal-102	21	F	1.034±0.015
Normal-103	40	F	0.870±0.017
Normal-104	48	F	0.312±0.025
Normal-105	48	F	1.722±0.070
Normal-106	47	M	2.334±0.070
Normal-107	41	F	0.235±0.012
Normal-108	45	F	0.961±0.011
Normal-109	37	F	0.997±0.035
Normal-110	49	M	1.935±0.048
Normal-111	51	F	1.491±0.050
Normal-112	31	F	0.612±0.058
Normal-113	50	M	0.787±0.057
Normal-114	41	M	2.254±0.034
Normal-115	50	M	0.977±0.021
Normal-116	45	M	1.182±0.033
Normal-117	59	M	1.181±0.056
Normal-118	41	M	1.043±0.036
Normal-119	47	M	1.465±0.075
Normal-120	45	M	1.154±0.079
Normal-121	40	M	2.240±0.087
Normal-122	37	M	0.872±0.028
Normal-123	37	F	0.731±0.013
Normal-124	39	M	1.501±0.043
Normal-125	51	M	1.210±0.024
Normal-126	62	M	2.461±0.163

Normal-127	47	M	1.402±0.066
Normal-128	46	M	1.200±0.044
Normal-129	58	M	1.528±0.046
Normal-130	30	F	1.686±0.034
Normal-131	49	M	1.167±0.107
Normal-132	40	M	1.252±0.048
Normal-133	55	M	0.856±0.042
Normal-134	53	M	1.684±0.113
Normal-135	40	M	1.149±0.036
Normal-136	44	M	1.315±0.038
Normal-137	45	F	0.276±0.026
Normal-138	40	F	0.602±0.032
Normal-139	66	M	1.710±0.087
Normal-140	68	M	1.124±0.049
Normal-141	43	F	0.569±0.072
Normal-142	41	M	1.409±0.092
CRC-1	51	F	0.518±0.067
CRC-2	58	M	1.449±0.025
CRC-3	57	M	0.567±0.017
CRC-4	52	F	1.994±0.112
CRC-5	58	M	1.573±0.035
CRC-6	63	F	1.576±0.076
CRC-7	65	M	0.875±0.030
CRC-8	64	M	1.109±0.035
CRC-9	66	F	0.905±0.009
CRC-10	67	F	2.247±0.063
CRC-11	58	F	1.082±0.031
CRC-12	74	M	0.506±0.008
CRC-13	57	F	1.645±0.053
CRC-14	62	M	2.418±0.074
CRC-15	67	M	1.904±0.066
CRC-16	58	M	2.153±0.098
CRC-17	48	F	0.970±0.042
CRC-18	45	F	0.812±0.011
CRC-19	82	M	0.768±0.020
CRC-20	78	F	1.783±0.065
CRC-21	62	M	2.080±0.078
CRC-22	73	F	2.475±0.103
CRC-23	52	M	1.031±0.022
CRC-24	48	M	0.641±0.010
CRC-25	69	M	1.627±0.098
CRC-26	74	M	1.057±0.013
CRC-27	53	M	1.595±0.080
CRC-28	83	F	1.662±0.072

CRC-29	71	M	0.778±0.078
CRC-30	70	F	0.684±0.014
CRC-31	58	F	2.206±0.135
CRC-32	57	M	0.676±0.016
CRC-33	82	F	0.883±0.029
CRC-34	66	F	1.179±0.039
CRC-35	74	M	2.348±0.149
CRC-36	62	F	1.393±0.050
CRC-37	54	F	1.373±0.092
CRC-38	53	F	0.574±0.011
CRC-39	31	M	1.192±0.014
CRC-40	76	M	1.128±0.076
CRC-41	49	F	1.031±0.780
CRC-42	25	M	1.138±0.127
CRC-43	68	M	1.060±0.038
CRC-44	69	M	1.994±0.091
CRC-45	69	M	4.370±0.281
CRC-46	76	M	0.966±0.024
CRC-47	61	M	3.567±0.216
CRC-48	63	M	1.895±0.112
CRC-49	69	F	1.469±0.030
CRC-50	67	M	2.541±0.116
CRC-51	76	F	2.990±0.016
CRC-52	67	F	1.253±0.030
CRC-53	65	F	2.511±0.069
CRC-54	78	F	0.754±0.005
CRC-55	34	M	3.657±0.062
CRC-56	55	M	0.753±0.024
CRC-57	42	F	1.307±0.020
CRC-58	64	F	2.162±0.057
CRC-59	77	F	2.584±0.060
CRC-60	50	F	1.850±0.063
CRC-61	72	F	0.835±0.043
CRC-62	52	M	3.224±0.060
CRC-63	67	M	1.954±0.037
CRC-64	67	F	2.302±0.193
CRC-65	84	F	2.016±0.030
CRC-66	52	M	1.955±0.050
CRC-67	77	M	0.782±0.011
CRC-68	60	F	1.167±0.022
CRC-69	58	F	1.738±0.036
CRC-70	67	F	2.750±0.205
CRC-71	53	F	1.284±0.018
CRC-72	87	M	2.826±0.081

CRC-73	45	F	1.644±0.067
CRC-74	77	M	1.346±0.036
CRC-75	65	M	0.998±0.031
CRC-76	57	M	2.362±0.069
CRC-77	65	F	2.531±0.051
CRC-78	53	M	2.571±0.084
CRC-79	57	M	2.075±0.056
CRC-80	82	M	0.914±0.032
CRC-81	50	M	3.262±0.068
CRC-82	85	M	1.296±0.046
CRC-83	83	F	1.577±0.029
CRC-84	73	M	4.103±0.077

Table S2. The optimized MS conditions used for the analysis of 8-OHdG.

Compound	MRM ion transition (*m/z*)	DP (V)	CE (V)	EP (V)	CXP (V)
8-OHdG	284.1>168.0	45.0	18.0	10.0	13.0
	284.1>117.0	45.0	22.0	10.0	5.0
[$^{15}N_5$]8-OHdG	289.1>173.0	45.0	18.0	10.0	13.0

DP declustering potential, *CE* collision energy, *EP* entrance potential, *CXP* collision cell exit potential
